# Lung Diseases and Rare Disorders: Is It a Lysosomal Storage Disease? Differential Diagnosis, Pathogenetic Mechanisms and Management

**DOI:** 10.3390/children11060668

**Published:** 2024-05-30

**Authors:** Chiara Montanari, Veronica Maria Tagi, Enza D’Auria, Vincenzo Guaia, Anna Di Gallo, Michele Ghezzi, Elvira Verduci, Laura Fiori, Gianvincenzo Zuccotti

**Affiliations:** 1 Department of Pediatrics, Vittore Buzzi Children’s Hospital, University of Milan, 20154 Milan, Italy; chiara.montanari@unimi.it (C.M.); veronica.tagi@unimi.it (V.M.T.); enza.dauria@unimi.it (E.D.); vincenzo.guaia@unimi.it (V.G.); anna.digallo@unimi.it (A.D.G.); michele.ghezzi@asst-fbf-sacco.it (M.G.); laura.fiori@asst-fbf-sacco.it (L.F.); gianvincenzo.zuccotti@unimi.it (G.Z.); 2 Department of Biomedical and Clinical Science, University of Milan, 20157 Milan, Italy; 3 Department of Health Sciences, University of Milan, 20146 Milan, Italy; 4 Metabolic Diseases Unit, Department of Pediatrics, Vittore Buzzi Children’s Hospital, University of Milan, 20154 Milan, Italy

**Keywords:** Lysosomal storage diseases, Gaucher disease, Acid sphingomyelinase deficiency, mucopolysaccharidosis, Pompe disease, lung, respiratory system, diagnosis, treatment

## Abstract

Pulmonologists may be involved in managing pulmonary diseases in children with complex clinical pictures without a diagnosis. Moreover, they are routinely involved in the multidisciplinary care of children with rare diseases, at baseline and during follow-up, for lung function monitoring. Lysosomal storage diseases (LSDs) are a group of genetic diseases characterised by a specific lysosomal enzyme deficiency. Despite varying pathogen and organ involvement, they are linked by the pathological accumulation of exceeding substrates, leading to cellular toxicity and subsequent organ damage. Less severe forms of LSDs can manifest during childhood or later in life, sometimes being underdiagnosed. Respiratory impairment may stem from different pathogenetic mechanisms, depending on substrate storage in bones, with skeletal deformity and restrictive pattern, in bronchi, with obstructive pattern, in lung interstitium, with altered alveolar gas exchange, and in muscles, with hypotonia. This narrative review aims to outline different pulmonary clinical findings and a diagnostic approach based on key elements for differential diagnosis in some treatable LSDs like Gaucher disease, Acid Sphingomyelinase deficiency, Pompe disease and Mucopolysaccharidosis. Alongside their respiratory clinical aspects, which might overlap, we will describe radiological findings, lung functional patterns and associated symptoms to guide pediatric pulmonologists in differential diagnosis. The second part of the paper will address follow-up and management specifics. Recent evidence suggests that new therapeutic strategies play a substantial role in preventing lung involvement in early-treated patients and enhancing lung function and radiological signs in others. Timely diagnosis, driven by clinical suspicion and diagnostic workup, can help in treating LSDs effectively.

## 1. Introduction

Recurrent respiratory symptoms and impaired lung function in childhood should be attended to and investigated by pulmonologists, with the goal of looking for an underlying disease. Lysosomal storage diseases (LSDs) are inherited metabolic disorders usually accompanied by pulmonary involvement [1]. In LSDs, a single gene mutation causes defects of an enzyme, an enzyme activator, or associated proteins, leading to the impairment of lysosome function and to the consequent substrates’ accumulation [2].

We will consider the following main LSDs with possible pulmonary involvement during childhood: Gaucher disease (GD), Acid sphingomyelinase deficiency (ASMD), Mucopolysaccharidoses (MPS) and Pompe disease (PD).

GD, the most prevalent inherited LSD [3], is an autosomal recessive disease due to mutations in the glucocerebrosidase gene (GBA), leading to lysosomal hydrolase acid β-glucosidase deficiency and accumulation of glucosylceramide in the lysosomes of macrophages (called Gaucher cells) [4,5,6]. The liver, spleen, bone marrow, and, in more severe cases, lung and kidney are the more involved organs [7]. Even though we distinguish three clinical phenotypes in GD, the spectrum of full disease is a continuum of signs and symptoms. Type 1 GD is the most common type in adult age, being responsible for around 90% of adult cases, but the onset can also be during childhood. The clinical phenotype is heterogenous, varying from asymptomatic to severe presentation with (epato)-splenomegaly, anaemia, thrombocytopenia, bone infarction with fractures, aseptic osteonecrosis, osteopenia, growth restriction and lung manifestations. In type 1 GD, neurological damage is absent, while it is present in types 2 and 3 [8]. Type 2 GD is the most severe form, with rapidly progressive neurological impairment since the first months of life. Splenomegaly is usually present. Type 3 GD is a chronic neuronopathic form with a heterogenous phenotype; visceral and bone disease could be present [9]. Pulmonary involvement is described both in type 2 and 3, with a higher risk in patients homozygous for the L444P mutation [7].

ASMD is a rare autosomal recessive lysosomal storage disorder caused by mutations in the SMPD1 gene, resulting in the accumulation of sphingomyelin in various organs [9]. ASMD is categorised into infantile neurovisceral, chronic neurovisceral and chronic visceral based on clinical phenotype and disease progression. Infantile neurovisceral ASMD typically presents within the first 6–10 months of life with severe neurological symptoms and visceromegaly, leading to death by around the age of 3 years. Chronic visceral ASMD follows a protracted, multi-systemic course with varied severity, often extending into adulthood with periods of stability. Chronic neurovisceral ASMD shares similarities with chronic visceral ASMD but with earlier onset and potentially mild neurological symptoms [10,11]. Without treatment, patients with chronic neurovisceral ASMD may survive beyond childhood, sometimes reaching adulthood [12]. Lung disease, hepatosplenomegaly, liver dysfunction, thrombocytopenia, coagulation defects, dyslipidemia, bone issues, and growth delays are the typical non-neurological symptoms of ASMD [13,14]. Lung involvement, especially interstitial lung disease (ILD), is more frequent in chronic visceral ASMD, although it may be found in all ASMD forms and may occur at all ages [15,16].

MPS encompass a group of rare genetic disorders affecting the catabolism of glycosaminoglycans (GAGs). Each disorder within the MPS spectrum is attributed to a deficiency in the enzymatic activity of a specific lysosomal enzyme that is essential for the degradation of GAGs. The pathogenesis is characterised by the progressive accumulation of partially degraded GAGs within the lysosomes [1]. The accumulation of GAGs has been observed to induce damaging effects on a multitude of tissues and organs, thereby giving rise to a range of clinical manifestations, including skeletal deformities, joint rigidity, growth retardation, coarse faces, corneal clouding, hearing loss, as well as compromised pulmonary and cardiac function [17]. Impairments in the central nervous system can also be present and associated with abnormal behavioural patterns. MPS disorders are classified into seven distinct types according to the specific enzyme deficiency and the subsequent accumulation of substrate in the affected individual (Table 1) [2].

PD was the first lysosomal storage disorder to be discovered. It has an autosomal recessive inheritance pattern. The pathogenetic mechanism consists of the deficiency of the enzyme acid alpha-glucosidase (GAA), which mediates the lysosomal degradation of glycogen into glucose. The deficiency of GAA leads to the accumulation of glycogen within the lysosome in all tissues. The pathologic activation of the inflammatory cascades that follow primarily affects skeletal and cardiac muscles [15]. The clinical spectrum of PD varies considerably in age of onset, severity of disease progression, and overall clinical phenotype. Although PD is a continuum of the disease spectrum, two general subgroups are identified in clinical practice according to the age of onset of symptoms and their severity [18]. Classic infantile-onset Pompe (IOPD) disease is the most severe phenotype, with a GAA activity of <1%. Life expectancy is 18 months if left untreated. It typically presents within 6 months of age, with hypertrophic heart disease leading to heart failure, hypotonia, diffuse muscle weakness, areflexia, increased serum levels of CPK, macroglossia, feeding difficulties, failure to thrive and cardio-respiratory failure [19]. Although cases of late-onset Pompe disease (LOPD) can occur during infancy, they usually manifest later in life due to some residual GAA activity [18,19]. LOPD is characterised by muscular weakness, myalgia and asthenia with exercise intolerance and elevated creatine kinase, with cardiological involvement; in LOPD, the respiratory disease typically presents with a restrictive pattern [20].

Lung function in patients with LSDs may be compromised through different mechanisms, all linked to the pathogenesis of the disease itself. LSDs include about 70 diseases which are individually rare but collectively affect 1 in 5000 live births [2]. Respiratory function can be impaired as a result of the storage of substrate in the lung parenchyma (alveoli and interstitium) with a consequent compromised alveolar gas exchange in muscle and bone with restrictive pattern and in the upper and lower airway with the obstructive pattern. Different kinds of pulmonary diseases can be present in a single disease, and different diseases can share similar multi-organ involvement with clinical overlapping [21]. Moreover, in some LSDs with neurological impairment, a respiratory dysfunction can be the consequence of the respiratory centre compromise [22], and in the case of a swallowing impairment, respiratory symptoms can be a consequence of chronic airway aspiration [20].

In some cases, recurrent respiratory symptoms could precede the specific clinical suspect and then the diagnosis [1].

The aim of the present review is to describe the above-mentioned LSDs with pulmonary involvement, focusing on the mild phenotypes which can be misdiagnosed and where respiratory problems may be one of the presenting symptoms. Pediatric pulmonologists may face a patient with pneumopathy as part of an underlying systemic disease. We will describe the clinical elements of differential diagnosis among these diseases, whose symptoms often overlap, in order to guide clinical suspicion and differential diagnosis. Afterwards, we will describe the specific management of each disease, including treatment and follow-up. Since new therapeutic strategies like enzymatic replacement therapy (ERT) seem to be useful in preventing lung disease or its progression, an early diagnosis is pivotal.

## 2. Methods

We conducted a narrative review to explore the typical clinical and radiological patterns of lung diseases in LSDs, their differential diagnosis and management in LSDs. We conducted an extensive literature search on PubMed (Medline) and Scopus databases. The language was restricted to English only, and publications from January 2000 to March 2024 were included. We included original research articles, systematic reviews, narrative reviews, guidelines, case reports and case series in our analysis. The research strategies for each described disease are reported in Table 2.

Starting from a total of 326 papers, 214 articles were excluded by a first screening on the basis of titles and abstracts. The authors then reviewed the full texts of the remaining papers and finally selected 112 relevant articles, which were analysed to provide a critical discussion. The reference list of all articles was checked.

**Figure 1:**
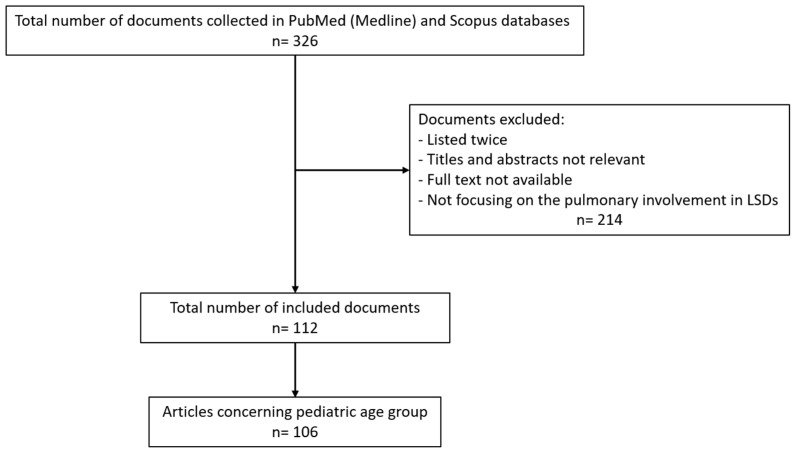
A flow chart shows the process of article selection and exclusion.

A diagram graphically showing the process of paper selection and exclusion is reported in Figure 1.

## 3. Results

### 3.1. Clinical Presentation

#### 3.1.1. Primary Respiratory Impairment

Lung parenchymal damage may be present in GD and in ASMD as the consequence of primary storage of exceeding substrates [2].

Gaucher’s cells infiltrate into the alveoli, interstitium, and bronchi, which can lead to respiratory symptoms, eventually leading to interstitial lung disease (ILD) and restrictive pneumopathy pattern [19,23].

In addition, storage into pulmonary vasculature can determine a pulmonary vascular disease and a pulmonary arterial hypertension in GD patients [19]. A pulmonary arterial hypertension in GD patients can also be a consequence of chronic hypoxaemia, due to ILD, and of associated liver disease with intrapulmonary shunting and hepatopulmonary syndrome, or in patients undergoing splenectomy [5,24,25,26].

Although a different pulmonary involvement has been reported even in monozygotic twins [27], recent evidence suggests a higher prevalence of ILD in patients with L444P homozygous mutations [27].

In ASMD, ILD is the main type of pulmonary pathology, which is more frequent in chronic visceral ASMD, although it may be found in all ASMD forms and may occur at all ages. The storage of Niemann–Pick cells in the alveolar septa and bronchial walls is the underlying mechanism [15]. ILD has a large variability in onset age, from the neonatal period to adulthood [16]. Up to 42% of patients present with dyspnea at diagnosis [28]. Pulmonary function impairment represents about 25% of the causes of death in patients with chronic visceral ASMD [29,30]. Nearly 82% of patients die because of ILD, associated with infectious pneumonia in 30% of cases or pulmonary embolism in 4% of cases [30].

Another not-so-frequent cause of lung impairment at the alveolar and interstitial level and pulmonary hypertension following vasculature involvement is MPS [31]. As regards the primary involvement of the lung parenchyma in MPS patients, a study suggested the presence of direct alveolar and interstitial pulmonary damage due to GAG storage. Neonatal interstitial lung disease is an atypical presentation for MPS I, which is likely under-recognized and should be considered in the differential diagnosis of patients with persistent respiratory distress not controlled by multiple surfactant therapies after ruling out the common etiologies [32]. A few case reports described respiratory distress syndrome related to neonatal onset interstitial lung disease in newborns with a genetically confirmed diagnosis of MPS I [33]. Primary lung involvement is not commonly observed in MPS III (Sanfilippo syndrome). However, a case report describes the case of a neonate exhibiting persistent tachypnea associated with parenchymal abnormalities observed on lung imaging; the infant was later diagnosed with MPS IIIA. This case suggests that patients with MPS may exhibit earlier modes of presentation and atypical findings [34].

Moreover, a study conducted on MPS IIIA mice showed how increased storage of heparan sulphate in mouse lung tissue, combined with decreased gene and protein expression of surfactant proteins, can lead to altered pulmonary surfactant synthesis in MPS IIIA mice. This could demonstrate that patients with MPS IIIA may present an impairment of surfactant composition and function. The presence of undegraded heparan sulphate in alveolar surfactant may modify the distribution and function of the interfacial surfactant film components, and this destabilises the interfacial film and inactivates surfactant, altering the ability of surfactant to regulate alveolar surface tension. The consequent alveolar disease can lead to an increased susceptibility of MPS IIIA patients to pulmonary infections and other respiratory issues [35].

Another possible cause of primary pulmonary involvement is airway impairment. Typically, airway respiratory disorders are observed in all patients diagnosed with MPS and manifest across all types of MPS. Both upper and lower airways may be involved. Respiratory manifestations are primarily the consequence of GAG accumulation, leading to initial upper airway obstruction, particularly affecting the oral cavity, nasal passages, and pharynx [25]. At lower airways, the progressive GAG storage leads to mucosal thickening and bronchial collapse with a possible obstructive respiratory pattern (asthma), as for the accumulation of dense secretions [21]. Tracheobronchial involvement and tracheobronchomalacia can be attributed to the accumulation of GAGs within the chondrocytes and extracellular matrix of the trachea, leading to acute symptoms as inspiratory stridor, with possible respiratory failure [21]. Lower airway involvement becomes more noticeable with increasing age and is most prominent in MPS I, II, IV and VI.

The clinical presentation of pulmonary involvement in patients with MPS is characterised by a range of symptoms. In lower airways disease manifestations include dyspnea, decreased clearance of secretions, persistent cough, wheezing, and recurrent episodes of bronchitis or pneumonia [36]. Upper airways manifestations include dyspnea, pronounced snoring coupled with obstructive sleep apnea, thick nasal discharge, excessive production of thick mucus secretions, and recurring episodes of sinusitis, otitis and tonsillitis [37].

In MPS I (Hurler syndrome), the main consequences of respiratory involvement are represented by recurrent upper and lower airway infections and upper airway obstruction during sleep, resulting in moderate or severe obstructive sleep apnea [38]. Also, in attenuated phenotypes of MPS I (Scheie and Hurler-Scheie syndrome), obstructive respiratory patterns are often observed [36]. A comprehensive analysis of the bronchoscopies performed in a cohort of 30 patients diagnosed with MPS II (Hunter syndrome) revealed a high prevalence of widespread GAG storage, commonly affecting the lower respiratory tract [39], causing trachea-bronchomalacia. Patients with MPS II may exhibit symptoms of hoarseness, stridor, and breathing difficulties [40]. Moreover, frequent infections of the upper respiratory tract are a common clinical feature observed in this group of patients [36,41]. In MPS IV (Morquio syndrome), the development of obstructive lung disease may occur as a consequence of various tracheobronchial abnormalities, and also in MPS VI (Maroteaux-Lamy syndrome), obstructive lung disease is related to the presence of narrowed bronchial airways and trachea-bronchomalacia, that can result in acute airway obstruction or collapse [42].

#### 3.1.2. Secondary Respiratory Impairment

In MPS, skeletal deformities may determine a restrictive respiratory pattern, especially in MPS type IV and VI, as demonstrated in patients’ pulmonary function tests [43]. Kyphosis, scoliosis, and kyphoscoliosis frequently encounter musculoskeletal conditions in MPS disorders. The augmentation of the anteroposterior diameter of the chest can cause the repositioning of the ribs into a more horizontally aligned configuration, leading to anomalies in respiratory mechanics. Pectus carinatum is observed to have a higher incidence in individuals diagnosed with MPS IV. The presence of an atypical thoracic structure, stiff costo-vertebral joints, and upward displacement of the diaphragm due to the eventual hepatosplenomegaly contribute to inadequate mechanical properties of the thoracic cavity. These multiple abnormalities in MPS patients result in a reduced ventilatory capacity, manifesting as a reduction in vital capacity (VC). Pulmonary function can also be affected by the characteristic short stature and skeletal dysplasia [39,44].

In MPS IV (Morquio syndrome), both upper and lower airway involvement, as mentioned above, and the typical anomalies of the skeletal system (short stature and thoracic cage deformity) can result in a mixed obstructive and restrictive pattern. In Morquio syndrome, as described in a study, a restricted chest cage can be responsible for the development of microatelectasis. This can lead to right-to-left shunting and consequent chronic hypoxemia, as demonstrated by pulmonary function tests [45].

Patients diagnosed with MPS VI (Maroteaux-Lamy syndrome) may exhibit both obstructive and restrictive lung disease. On the other hand, restrictive lung disease is primarily attributed to a small and stiff thoracic cage, accompanied by abdominal distention, kyphosis, scoliosis, and increased lumbar lordosis [36]. Children with MPS VI have decreased pulmonary function and reduced endurance, and patients older than ten years may progress to severe pulmonary obstruction and respiratory failure requiring tracheostomy [46]. Also, in MPS IX (Natowicz syndrome), progressive craniofacial and skeletal abnormalities may lead to progressive respiratory impairment [19,47]. In MPS II, other physical characteristics, including anomalies in the shape and structure of the ribs, enlargement of abdominal organs, short neck, and restricted mandibular mobility, contribute to worsening respiratory problems [36,41].

Furthermore, an exacerbation of ventilatory compromise during sleep has been observed, and sleep obstruction pattern disorders have been well documented in individuals diagnosed with MPS [19], either because of the upper airway obstruction by GAGs storage or because of central-type apnea caused by spinal cord compression, increased intracranial pressure and brainstem’s sleep regulatory mechanism impairment. Moreover, central apnea can be the consequence of dysfunction of the respiratory centre in the medulla, as described in patients with MPS II. Typical polysomnographic findings in MPS patients include the presence of OSA, recurrent arousals, reduced REM sleep, and O2 desaturation. In MPS III (Sanfilippo syndrome), abnormal breathing patterns can occur as a consequence of neurodegenerative processes and not as a primary pulmonary involvement [48]; neurocognitive decline can be one of the main factors in the pathophysiology of sleep problems [22,44]. Moreover, the presence of viscous secretions, along with inadequate clearance mechanisms, can contribute to obstructive patterns [48].

In LOPD, musculoskeletal involvement affects respiratory function; first of all, respiratory failure due to muscle weakness is very common in PD; the earlier the diagnosis, the more severe the damage. Infants with IOPD present muscle hypotonia and early cardio-respiratory failure, which impacts morbidity and mortality within the first year of life if treatment is not received. LOPD patients, on the other hand, exhibit a progressive decline in respiratory function with delayed onset of symptoms [49]. LOPD is characterised by a restrictive phenotype related to respiratory muscle weakness, primarily the diaphragm [50]. The onset of diaphragm muscular impairment can precede limb muscle weakness. In PD, ventilatory failure remains a major cause of morbidity and mortality in the absence of treatment [49].

Moreover, scoliosis is a complication often described, contributing to the restrictive pattern; in these patients, pulmonary function is consistently reduced, requiring respiratory support [49].

In PD, pulmonary symptoms can have an infective origin, as weakness of expiratory muscles prevents effective coughing [51].

The upper respiratory airways are involved in the clinical manifestations of PD as well. Specifically, the tongue is universally affected in patients with PD, leading to muscular weakness and macroglossia. This implies an impaired ability to maintain the larynx patent, especially during sleep, leading to obstructive sleep apnea [51].

Moreover, patients may exhibit a variety of other manifestations, including a narrowed nasal tract and oral cavity, a reduction in the vocal folds’ movement, dysphagia, and subsequent aspiration due to poor oropharyngeal control. These findings were consistently found in patients with IOPD despite early initiation of treatment [52]. In LOPD, patients’ weakness in the diaphragm and respiratory muscles can lead to an impaired cough and airway clearance as well as hypoventilation throughout the night with sleep-related breathing issues [49].

In PD, the CNS is not spared by the lysosomal dysfunction. Indeed, glycogen accumulation was found to be present in the anterior cervical spinal cord, associated with neuronal swelling [53] and in the brainstem neurons [54]. Lastly, phrenic motor-neurons were also found to have swollen cell bodies [55]. While the respiratory muscle dysfunction typical of PD is found to be related to central neurological damage as well, the role of the involvement of brainstem respiratory networks has not been evaluated [56]. Thus, sleep apnea in these patients may be both obstructive and central [56].

Furthermore, in GD, hepatosplenomegaly and spinal deformities may also contribute to the pathogenesis of restrictive pulmonary disease [22]. Hepatosplenomegaly can lead to the same in ASMD.

### 3.2. Approach to Differential Diagnosis

**Figure 2:**
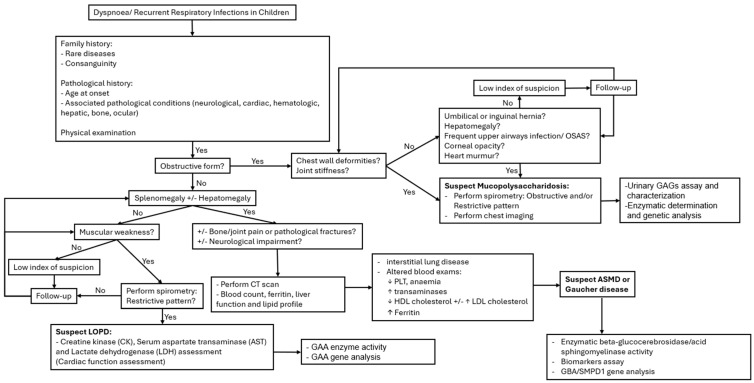
Approach to differential diagnosis.

Respiratory manifestations associated with rare inherited metabolic disorders may be part of a constellation of symptoms indicating an underlying multisystemic disease, such as LSDs. Mild phenotypes of LSDs, characterised by late onset and less severe organ involvement, may be misdiagnosed or remain undiagnosed. A pulmonologist should be able to raise suspicion of a rare disease through history and objective examination when faced with a patient exhibiting recurrent respiratory symptoms. A flow chart outlining the main clinical signs and recommended investigations for the differential diagnosis of LSDs is provided in Figure 2.

Familial medical history should be investigated, with particular attention to consanguinity, the presence of rare diseases or neurological impairments, or instances of death during the first years of life. The patient’s personal medical history should evaluate neonatal history, age of onset of respiratory symptoms, and the presence of other medical issues such as cardiac, neurological, hepatic, haematological, bone pain or pathological fractures, osteoarticular problems, ophthalmic involvement, snoring, and nocturnal apnea.

During physical examination, characteristics of respiratory symptoms such as the presence of an obstructive component in the lower airways (e.g., asthma) or other obstructive symptoms like stridor in the upper airways should be assessed. Skeletal abnormalities, especially in the chest, should also be investigated. Subsequently, examination of other organs should be conducted, searching for the presence of hepatomegaly or splenomegaly, heart murmurs, hernias, corneal clouding, and signs of asthenia with reduced strength. Moreover, vital signs, body weight, and height measurements should be assessed during physical examination [20]. If a rare disease is suspected, diagnostic investigations should be discussed with a multidisciplinary team, including paediatricians, radiologists, anesthesiologists, otolaryngologists, as well as specialists such as cardiologists, ophthalmologists, hepatologists, and neurologists (see Figure 2).

Typically, a chest X-ray is the primary method used to rule out pulmonary involvement and determine the type and severity of impairment. Since ILD is common, it is advisable to use high-resolution chest CT (HRCT) to assess the extent and severity of lung abnormalities as second-level imaging. Spirometry is the first level functional exam that is useful to evaluate an obstructive pattern, in case of bronchospasm and lower airway involvement, or a restrictive picture secondary to chest wall deformities or muscular weakness. The diagnostic work-up may also include bronchoscopy and bronchoalveolar lavage in cases of interstitial pneumonia and DLCO, if applicable for age.

#### 3.2.1. Radiological and Lung Function Patterns

According to the pulmonary clinical presentation, we will find typical radiological signs and anomalies in lung functionality exams.

Radiological findings are similar in GD and chronic visceral ASMD with ILD; on chest X-ray, diffuse interstitial and nodular infiltrates, with honeycombing, are described [20]. High-resolution computed tomography (HRCT) shows a typical pattern: ground-glass opacities, sometimes superimposed with interlobular and intralobular septal thickening, are the most frequent findings [11,57]. Patients with GD develop alveolar and interstitial opacities, with capillary obstructions and recurrent infections. Furthermore, pulmonary fibrosis is described with military pattern and mediastinal lymphadenopathy in GD patients [7]. Ground-glass opacities are usually predominant in the upper lung area in ASMD [11], where intralobular lines, overlapped with ground glass and interlobular septal thickening, create a “crazy paving pattern” [58,59]. Cysts, centrilobular nodular opacities, segmental atelectasis, bronchiectasis, and emphysema are rarely reported [58,59]. Respiratory functional tests reveal a restrictive pattern both in GD patients with ILD [60] and in about half of patients with chronic visceral ASMD [22,28].

Reduced diffusion capacity for carbon monoxide (DLCO) has been documented in GD patients with ILD [60] as well as DLCO is impaired in 75% of chronic visceral ASMD patients, being lower in patients younger than 18 years [22,28].

In ASMD, lung involvement can be confirmed by the presence of increased cell numbers and large multivacuolated histiocytes containing granules-stained deep blue with May–Grunwald–Giemsa (MGG) stain (Niemann–Pick cells or “sea blue” histiocytes) [15]. When lung biopsy is performed, several finely vacuolated foamy macrophages are observed inside the alveolar lumen and inside the alveolar walls [15,22], but also in the lymphatic interlobular vessels and the sub-pleural spaces [20].

When bronchoalveolar lavage fluid analysis and lung biopsy are performed, lipid-laden macrophages and foamy histiocytes in alveoli, interstitial tissue and subendothelial area are described in GD [60].

Although not essential for the diagnosis, bronchoalveolar lavage fluid analysis and lung biopsy may be helpful to confirm a pulmonary involvement in GD and in ASMD, showing foamy cells or foamy macrophages/cells [19].

In PD, due to the respiratory muscle weakness typical of PD, pulmonary function testing will demonstrate a restrictive pattern; total lung capacity will decrease as the disease progresses. Specifically, the forced vital capacity (FVC) will be reduced due to the involvement of the diaphragm, which is the main inspiratory muscle. To assess the degree of diaphragmatic weakness, spirometry may be performed both in the supine and the orthostatic position. A postural difference in FVC more than 10 signals a diaphragmatic weakness [61]. Monitoring respiratory function via pulmonary function testing in patients with PD should be part of the patient’s plan of care, independent of the symptoms recorded. Indeed, severe respiratory insufficiency is an important cause of morbidity and may ensue before any signs of limb-girdle muscle weakness [62]. Moreover, respiratory muscle strength may be measured directly via manometry or via trans-diaphragmatic pressure measurement [49]. Lastly, phrenic nerve conduction was studied via electromyography in patients with LOPD to assess a neurogenic component in diaphragmatic weakness; however, data were inconclusive, and phrenic nerve involvement was not confirmed [63]. As far as imaging is concerned, magnetic resonance imaging (MRI) is able to identify fatty infiltration of muscular tissue, both at the level of the respiratory muscles (paraspinal, abdominal and thoracic muscles) and also at the level of the tongue. Ultrasound may also be used to monitor these muscle groups. In the pediatric population, hyperpolarised 129Xe for functional pulmonary MRI (XeMRI) has recently been introduced for the assessment of ventilation and gas exchange. Lastly, fibroscopy should be performed in order to detect upper airway involvement, including oropharyngeal and laryngeal abnormalities [52].

In MPS, patients’ airways and skeletal imaging are utilised as valuable diagnostic tools in defining the aetiology of respiratory manifestations and assessing the level of the airway, skeletal and thoracic cage involvement [64,65].

CT analysis revealed noteworthy alterations in the morphology of the trachea, with a U-shaped configuration due to an aberrant accumulation of substances within the submucosal layer [66]. Another study was carried out involving a cohort of 22 individuals diagnosed with MPS IV. The findings of this study revealed a significant prevalence of a distinctive tracheal narrowing characterised by a triangular shape accompanied by an augmented degree of tracheal tortuosity in patients affected by MPS IVA [64]. The radiographic examination of the chest in MPS patients with respiratory involvement due to skeleton abnormalities reveals the presence of widened ribs exhibiting an “oar-shaped” appearance, along with a shortened thoracic region [39,44]. In MPS II, a compromised respiratory function is revealed by pulmonary function tests, specifically spirometry and plethysmography: a reduction in various parameters such as FVC, total lung capacity (TLC), and the ratio FEV1/FVC, indicating the presence of obstructive pulmonary impairment [39]. A broad single-centre study used an ultrasonic spirometer in a cohort of 35 patients with MPS with a medical history of respiratory problems to assess lung function. The findings of this study revealed that a vast majority of the subjects (91%) had small airway disease (FEF25–75% <65%), approximately half (48%) had restrictive lung disease, and a minority (9%) had obstructive lung disease [67].

A retrospective study was conducted using non-invasive pulmonary tests (impulse oscillometry system and/or pneumotachograph technique and thoracoabdominal motion techniques) to evaluate 17 patients with skeletal dysplasia, including 4 with Morquio syndrome (MPS IV). Patients with Morquio syndrome showed abnormal lung reactance, which is likely related to restrictive lung disease. These non-invasive pulmonary tests can be useful diagnostic tools in monitoring lung, airway and chest wall state in patients with Morquio syndrome who are not able to perform standard pulmonary function tests [68]. Another study conducted by Lin et al. described the results of pulmonary function assessment in a cohort of 35 pediatric patients diagnosed with mucopolysaccharidosis (MPS) and revealed the presence of either a restrictive or obstructive respiratory pattern [67]. Parenchymal abnormalities associated with airway obstruction have the potential to impair pulmonary oxygen uptake and carbon dioxide excretion [31,69].

The most common complications include an increased susceptibility to develop respiratory infections and persistent or recurrent focal atelectasis [70].

Nocturnal Hypoventilation should be suspected upon the appearance of symptoms such as de headache and daytime fatigue, which are related to CO2 retention, oxygen desaturation and hypercapnia, which can be assessed by emogasanalysis. In these cases, polysomnography should be performed to test for OSA [71].

#### 3.2.2. Clinical Manifestations Associated

In the context of the different molecular pathways involved, it is significant that some clinical presentations show similarities among the various LSDs. This observation implies that the accumulation of substances within lysosomes, causing subsequent cellular toxicity, leads to a similar outcome.

A crucial aspect in the differential diagnosis of respiratory involvement in patients with LSDs is to understand and establish the different pathologic mechanisms that lead to respiratory impairment in LSDs: infiltration of lung parenchyma by abnormal cells leading to interstitial lung involvement is observed in GD and ASMD; diaphragmatic and muscular weakness of the thoracic cage is a typical feature of LOPD, which is responsible for the rise of ventilatory distress with chronic respiratory failure; airway soft tissue infiltration, with consequent airway narrowing, accompanied by limited chest mobility as a result of skin and ligament stiffness, is observed in several types of MPS disorders; moreover in patients with MPS sleep-disordered breathing represents another common finding. Respiratory function could be worsened by other characteristic features of LSDs such as spinal and skeletal deformities (observed in GD, LOPD disease and MPS) and hepato-splenomegaly or isolated splenomegaly or hepatomegaly (observed in GD, ASMD, MPS and LOPD) that can compress the lungs and reducing their expansion, and cardiac involvement (observed in MPS and rarely in ASMD). In IOPD, cardiac involvement is a main feature of the disease, with high mortality caused by cardio-respiratory failure; thus, we consider LOPD mainly in differential diagnosis, where cardiac involvement is rare [72]. Respiratory symptoms constitute a common problem in patients with LSDs and are responsible for a number of clinical evaluations and complications, such as respiratory function decline [21,36]. Both in GD and ASMD, the most common respiratory symptoms are recurrent lung infection and dyspnea [57]. In LOPD, orthopnea, sleep apnea, effort dyspnea, exercise intolerance and respiratory infections are symptoms often described [72]. In MPS, recurrent respiratory infections can involve upper and lower airways, with persistent cough, wheezing, and episodes of bronchitis or pneumonia [36]. Skeletal deformities can also contribute to reduce the pulmonary function.

Although some clinical overlap, we can distinguish LSDs for some clinical signs and symptoms specifically associated. Table 3 summarises the main non-respiratory clinical manifestations of the analysed LSDs.

Three clinical subtypes of GD have been identified based on the existence or lack of neurologic symptoms (types 2–3 and 1, respectively) [57]. The typical non-neurological manifestations are (epato)-splenomegaly, anaemia, thrombocytopenia with easy bruising and bleeding, bone pain caused by infarction, aseptic osteonecrosis, osteopenia and fractures and bone marrow infiltration [8]. Lung involvement is an uncommon presentation symptom [57]; however, lung disease is described in all Gaucher disease types [20]. The infiltration of lipid-laden macrophages (Gaucher cells) into the alveolar, interstitial, perivascular, and peribronchial regions causes respiratory issues. Hepatopulmonary syndrome, also known as arteriovenous shunting, can also be seen as a consequence of chronic liver illness with consequent pulmonary hypertension [57]. Even though capillary storage by Gaucher cells may lead to pulmonary hypertension, the role of a circulating vasoactive substance has been postulated. Moreover, chronic hypoxemia secondary to ILD and pulmonary emboli derived from long bone fracture may contribute to pulmonary hypertension development, and digital clubbing is described as a sign of pulmonary vascular disease in GD [20]. Moreover, spinal abnormalities and hepatosplenomegaly, reducing diaphragmatic and chest excursion, reduce lung volumes leading to hypoventilation. As mentioned above, cases of swallowing disorders associated with neurological involvement lead to aspiration pneumonia [20].

ILD is present in most patients (>80%) with ASMD with a restrictive pattern disease. Clinical pulmonary manifestations often described are recurrent respiratory infections, including pneumonia and shortness of breath, but patients may also be asymptomatic for years. In these patients, pulmonary crackles are often missing or have ventral localisation [22]. However, patients with typical radiological features of ILD don’t have abnormalities in pulmonary function, so there isn’t a correlation between imaging and pulmonary function [5,12]. ILD, without treatment, can lead to respiratory insufficiency and oxygen need. Pulmonary hypertension was also described [12]. In infantile neurovisceral form chest radiography, ILD evidence was reported less frequently than chronic neurovisceral and chronic visceral. In case of neurological deterioration, bronchitis or aspiration pneumonia with the possibility of fatal events were reported. In patients with chronic visceral ASMD, only some cases of fatal lung disease are described [20].

In ASMD, the most common initial manifestation is hepatosplenomegaly, usually observed in childhood [13]. As described before, the clinical spectrum is variable in terms of age of onset, degree, and type of organ involved. The central nervous system is severely impaired in infantile neurovisceral form and variably in chronic neurovisceral form, with onset later in life and not rapidly progressive, ranging from mild hypotonia or hyporeflexia to loss of motor ability and mental impairment [73]. Patients with chronic neurovisceral form exhibit significant phenotypic heterogeneity in comparison with neurovisceral form [73], and other organs may be involved with different grades of severity. Thus, ASMD is a multisystemic disease with possible involvement of the heart, with left ventricular hypertrophy, conduction abnormalities and valvopathies, of bones, joint/limb pain and growth delay, and ocular, with cherry-red maculae [73]. Hepatic involvement can lead not only to hepatomegaly but also to an increase in transaminases and progressive liver cell damage [74]. In chronic neurovisceral and chronic visceral ASMD forms without a diagnosis, respiratory manifestations may be the reason for an early evaluation by a pulmonologist. HRCT should be recommended if suspected of pulmonary disease [5]. Cases of patients who underwent lung biopsy because of severe lower airway infections of unknown aetiology are described, permitting diagnosis of ASMD type B [5,57].

ASMD and GD have overlapping signs and symptoms, but generally, bone involvement is more characteristic of GD, while pulmonary disease, respiratory symptoms, and liver damage, with possible cirrhosis and liver failure, are more described in ASMD [74]. The bone marrow evaluation could differentiate ASMD, where lipid-laden foamy cells or cells with vacuolated cytoplasm are typical, from GD, where large cells with an eccentric nucleus and striated cytoplasm are present [1]. In case of suspect enzymatic evaluation of both beta-glucocerebrosidase and sphingomyelinase activities, it could be the quickest mode.

As described above in MPS, the combined restrictive and obstructive patterns, possibly associated with hypoventilation due to a typical neurological impairment in these patients, may lead to respiratory failure with an increased risk of infections. The progressive accumulation of GAGs e, with a multiorgan involvement, leads to organ failure and a consequent reduced life expectancy [43]. MPS is characterised by progressive and multi-systemic clinical manifestations. Frequent respiratory infections, otitis, hernias and organomegaly can present symptoms in the first 6 months in children with severe forms of MPS, especially in MPS I (Hurler Syndrome), which has the earlier onset [75]. Subsequently, gibbous, heart murmur, hypotonia and growth delay can appear from 6 to 12 months [76]. A typical coarse face is described, and macrocephaly becomes gradually more evident. MPS II, VI and VII may have a similar early phenotype [75]. There are three forms of MPS I; Hurler is the most severe, with significative cognitive impairment, multiple organs involved and precocious death in the first decade of life without therapy [75]. Hurler-Scheie and Scheie are attenuated forms, as a continuum of disease severity, where central nervous system impairment can be mild or absent, respectively, and the onset of symptoms is later and with better life expectancy [76]. In MPS I, growth is impaired by 3 years of age, while MPS II has a typical overgrowth in the first year of life, slowing after 5–6 years [77]. MPS III is characterised by a prevalent neurological involvement and is the most frequent type; somatic anomalies are less severe compared to behavioural problems, progressive cognitive decline and eventual epilepsy [75]. On the other hand, MPS IV and VI have exclusive somatic involvement, sparing the central nervous system. MPS IVA is characterised by stopping growth at 7–8 years of age and, contrary to the other MPS, by laxity and flexibility of joints, even though hips and shoulders may be stiffer. In MPS IVB, short stature is less severe, but inguinal hernia, hepatomegaly, hearing loss, corneal opacities, and frequent respiratory infections have been described, in addition to skeletal dysplasia [78]. Generally, in attenuated forms of MPS, the phenotypic spectrum is characterised by a later onset and a less fast progression. Excepting MPS III, symptoms are generally somatic without significant cognitive impairment. Dysmorphism may be mild. The typical clinical manifestations in mild forms are osteoarticular anomalies (joint stiffness and contracture, without inflammation signs, especially at the hands, carpal tunnel syndrome and mild multiple dysostosis), valvopathy, hernias, hepatomegaly, corneal clouding and upper and lower airway obstruction with recurrent infections [79].

In PD, due to muscle weakness, coughing may be ineffective, resulting in respiratory tract infections. Moreover, respiratory symptoms include sleep-disordered breathing with an evolution toward nocturnal hypoventilation, particularly pronounced in the supine position. Sleep disorders result in reduced quality of life by causing daytime sleepiness and fatigue. The spectrum of phenotypic manifestations of LOPD comprises bulbar muscle involvement manifesting as lingual weakness with dysarthria and dysphagia, osteoporosis, scoliosis, rigid spine syndrome (RSS), small-fibre neuropathy (SFN), sensorineural hearing loss, cardiac hypertrophy or abnormal cardiac rhythm, impaired gastric function and GI motility, lower urinary tract (LUT) and anal sphincter involvement, pain and fatigue [18].

#### 3.2.3. Biochemical Abnormalities

In GD thrombocytopenia is a common biochemical feature and it is secondary to hypersplenism and/or marrow infiltration. Anaemia or leukopenia, secondary to spleen status, may be present [22]. Liver enzymes may be slightly increased and cholestasis may be present [80]. Hyperferritinemia is a typical GD biochemical sign [81].

Clinical suspicion of GD can be confirmed by assay of enzymatic beta-glucocerebrosidase activity in leukocytes or by the presence of Gaucher cells in bone marrow aspiration, lastly confirmed by GBA gene mutation analysis [8]. Moreover, beta-glucocerebrosidase deficiency leads to glucosylceramide (GL1) accumulation. Its deacylated lysolipid, glucosylsphingosine (lyso-GL1), is considered a biomarker of GD, which can corroborate the diagnosis, evaluate response to treatment and diagnose GD caused by Saposin C deficiency, an activator for glucocerebrosidase [5,6]. Other key biomarkers are chitotriosidase, angiotensin-converting enzyme (ACE) and tartrate-resistant acid phosphatase (TRAP), which are elevated in GD [80].

In ASMD, low platelet cell count, as well as anaemia and leukopenia, are described. Dyslipidemia is typically associated with ASMD; specifically, low high-density lipoprotein-cholesterol levels are commonly associated [12]. Also, large spleen size is associated with secondary hypersplenism and risk of bleeding and bruising because of thrombocytopenia; however, no significant correlation between spleen volume and platelet count was found [73]. Another biochemical sign could be the consequence of liver dysfunction [73], as transaminases increase and other biochemical markers of cytolysis, liver cell damage, and cholestasis [74]. Numerous plasma biomarkers exhibit abnormally elevated levels in ASMD. Although some lack specificity, they serve as a reliable initial screening before assessing ASM enzyme activity, either independently or in conjunction with enzyme activity measurement. Among these biomarkers, sphingosylphosphorylcholine (SPC), also known as lysosphingomyelin (lyso-SM), stands out as the most specific. Combining its measurement with that of N-palmitoyl-O-phosphocholine-serine (PPCS), previously referred to as lysosphingomyelin-509, offers optimal results, particularly when enzyme activity assessment is conducted on dried blood spots [12]. However, the increase in chitotriosidase activity, a human chitinase secreted by macrophages, observed in ASMD is generally mild, inconsistent, and does not allow a differential diagnosis between ASMD and Niemann–Pick type C (NPC) or other diseases characterised by hepato-splenomegaly [12].

The diagnosis is lastly confirmed by SMPD1 gene mutation analysis [12].

In MPS, generally, no pathognomonic biochemical abnormalities are described, except for the pathological increase of urinary GAGs. Urinary GAGs can help in the diagnosis; however, the sole quantification can lead to false positives or false negatives. A complete procedure which includes both urinary GAGs quantitative and qualitative assay is recommended for the diagnosis of MPS in a clinically suspected population. Ascertaining the patient’s MPS type and subtype using lysosomal enzyme testing is necessary for a final, conclusive diagnosis [82]. Other biochemical abnormalities may be the consequence of the different organ involvement, but they are specific. Increased transaminases and secondary hypersplenism with decreased platelet counts may be seen in the case of hepato- and splenomegaly, respectively. An increase in bone remodelling enzymes (alkaline phosphatase, parathormone, etc.) with altered calcium-phosphorus metabolism may be present following skeletal involvement with impact on growth [82]. The diagnosis needs confirmation through the corresponding enzymatic determination and genetic analysis [22].

In LOPD laboratory testing, elevated serum muscle enzymes (specifically creatine kinase), elevated LDH, and AST may be found. If a blood smear is performed, vacuolated lymphocytes may be found. Serum creatine kinase (CK), a biomarker of muscle damage, is usually elevated in LOPD. CK elevations can be mild to moderate (e.g., 1.5 to 15 times the upper limit of normal). The onset of CK elevation may indicate the onset of clinical disease in presymptomatic patients or may begin to raise suspicion of LOPD before the onset of muscle weakness. In the case of cardiomyopathy, it may be seen upon electrocardiographic investigation with a short PR interval and increased height of QRS complexes diffused in all leads. Electrodiagnostic studies may also be abnormal, with evidence of fibrillations, complex repetitive discharges, positive sharp waves, and myotonic discharges upon paraspinal sampling [83]. The definitive diagnosis of PD is made by the determination of GAA enzyme activity in leukocytes in case of clinical suspicion, followed by GAA molecular analysis [19].

### 3.3. Treatment and Follow-Up

Timely diagnosis and treatment of lysosomal storage disorders (LSDs) play a crucial role in preventing irreversible lung damage. Early initiation of ERT, available for several LSDs, has been shown to at least partially prevent organ failure [19].

Simple, specific management strategies can greatly improve the quality of life and life expectancy for these patients. For instance, in LSDs with skeletal abnormalities, ensuring appropriate seating with trunk support for children and making periodic adjustments during growth can help mitigate the development of scoliosis, resulting in restrictive lung disease [2].

A personalized physiotherapy regimen should be maintained consistently. Close monitoring of nutrition and vaccination status is essential, and bronchoscopy may be necessary for removing bronchial casts [57].

#### 3.3.1. Gaucher Disease

Given the significance of lung complications in GD, it’s crucial to refer patients to pulmonary specialists and schedule regular monitoring at predetermined intervals. Enzyme replacement therapy (ERT) with imiglucerase, velaglucerase, or taliglucerase is now accessible for treating GD [84]. These medications have significantly reduced the occurrence of early-onset breathing difficulties and lung failure in youths. However, in rare instances that have been reported, ERT has not demonstrated efficacy in treating ILD [84,85].

Four cases of lung transplantation in patients with GD, two for ILD and two for pulmonary hypertension, have been reported. Notably, one child underwent lung transplantation at the age of 10 and experienced successful treatment outcomes, with no signs of ILD three years later [86].

There’s a suggestion to integrate lung function and chest imaging findings into the GD severity score to help physicians assess lung health and evaluate the effectiveness of treatment in GD.

Since GD patients not receiving ERT may face an increased risk of aspergillosis or mycobacterial infection, screening for opportunistic pulmonary infections and targeted treatment is important [87].

Splenectomy should be avoided in GD patients due to its association with pulmonary hypertension development [26,88].

Patients with progressive pulmonary disease may require long-term oxygen therapy [19].

#### 3.3.2. Acid Sphingomyelinase Deficiency

Since ILD is common among individuals with ASMD, particularly in younger patients, it is recommended to conduct baseline chest X-rays and high-resolution chest CT scans, with periodic follow-ups as necessary, to monitor this condition [12]. CT is the preferred imaging method to assess interstitial lung disease, often revealing a characteristic ground glass appearance. Pulmonary function tests, particularly measuring the DLCO, and exercise testing are valuable for identifying impaired gas exchange [89].

Pulmonary dysfunction is a hallmark of ASMD, affecting most patients, some of them presenting with progressive deterioration and respiratory failure [21,30,90]. However, some adults with attenuated ASMD may show no lung disease symptoms [91].

DLCO serves as a meaningful measure of disease burden in ASMD patients [21]. However, due to the rarity of ASMD and limited diagnostic screening, there remains a scarcity of evidence, particularly regarding mortality/survival rates and the frequency and timing of significant clinical events [11,12,21].

Olipudase alfa is now offered as ERT for the treatment of the non-neurological manifestations of ASMD, yielding promising outcomes [90,92]. In a 52-week randomised controlled trial involving 36 adult patients worldwide, the treatment group exhibited a significant absolute mean increase in DLCO of 22%, compared to 3% in the placebo group [93]. An open study involving 20 children with ASMD (ASCEND-Peds) revealed a mean DLCO increase of 33% after 52 weeks in 9 patients [93]. Additionally, three instances of lung transplantation in ASMD-B have been documented [94,95,96]. Among these cases, one patient passed away 29 days after transplantation, another was discharged after 80 days, and the third remained alive 35 months following the lung transplantation [19,94,95]. However, lung transplantation is associated with a high risk of complications due to potential disease recurrence and the release of ceramide during the debulking process after cleavage of the sphingomyelin substrate by transplanted lung cells, which may trigger a pro-inflammatory cascade, similar to the effects observed in ERT [19,97].

Splenectomy is not recommended in acid sphingomyelinase deficiency (ASMD) due to its potential exacerbation of pulmonary disease [28], except in cases of extensive spleen necrosis [97].

#### 3.3.3. Mucopolysaccharidosis

ERT is a treatment option available for several types of mucopolysaccharidosis (MPS) disorders, including MPS types I, II, IV, and VI. This treatment’s benefits include notable enhancements in walking ability, respiratory function, and enhanced quality of life. Hematopoietic stem cell transplantation (HSCT) has demonstrated its potential in preserving cognitive function and ex-tending overall survival in children affected by the most severe manifestation of MPS I. Furthermore, ongoing studies are currently exploring the applicability of HSCT in other MPS disorders [98,99].

Improved pulmonary function is observed in patients with MPS who undergo ERT, and this is likely attributed to a variety of mechanisms, including a decrease in the accumulation of GAGs within the airways, as well as an augmentation in chest wall compliance, respiratory muscle strength and endurance, and diaphragmatic excursion. Additional investigation is needed to ascertain the specific mechanisms that are pre-dominantly impacted by enzyme replacement therapy in relation to the improvement of pulmonary function of MPS patients [67,100].

A comprehensive study found that children with MPS VI showed improvements in their respiratory function following 72–96 weeks of ERT, with increases in FVC and FEV1 of 17% and 11%, respectively, over baseline measures. The FVC (%) rate did not change after 3.5 years of ERT treatment, according to phase III clinical trial results of ERT for MPS I, showing clinical improvements at six months of 5.6% compared to the placebo group [101].

In one study, patients with mild forms of MPS II and MPS VI who had received ERT for 1.5–7.4 years underwent spirometry as a follow-up test. The FVC and FEV1 measures in these patients increased from their pre-treatment levels. The respiratory system was more severely impaired in post-pubertal and pubertal patients than in pre-pubertal patients. A worsening of obstruction as a result of progressive tracheobronchomalacia may be the reason for the negative association observed between age-advancing and respiratory system function impairment [102].

Utilising spirometry and oximetry parameters as reference criteria, a longitudinal post-therapeutic study was conducted on patients with MPS IVA to assess the effects of ERT on clinical outcomes and respiratory function. Additionally, the study examined the longitudinal effects of adenotonsillectomy and NIV. According to the study’s findings, pulmonary function generally declined over time, as seen by a decrease in the spirometry parameters FEV1 [%Pred] and FVC [%Pred]. Oximetry tests showed the greatest improvements when ERT was used, indicating that it may slow down but does not stop the normal course of respiratory failure. The study’s findings indicate that the non-ERT group experienced a more marked decline in pulmonary function, whereas the ERT group experienced a more attenuated decline, although without any evidence of a reversal of the condition. This suggests that the early start of ERT prevents GAG accumulation rather than treating it. Furthermore, this study showed that for patients receiving ERT, established adjuvant therapies such as adenotonsillectomy and NIV seem to work better, either improving respiratory function or slowing the deterioration [103].

Disease-targeting therapies for MPS, such as enzyme replacement therapy (ERT) and hematopoietic stem cell transplantation (HSCT), may also benefit sleep-disordered breathing by lowering GAG storage in the upper airway, albeit there are limited data and evidence to support and confirm this. Early intervention may also stop anatomical anomalies from developing, which could lead to restrictive lung disease or upper airway obstruction. There is, however, little research assessing how ERT or HSCT affect sleep-disordered breathing [22].

#### 3.3.4. Pompe Disease

Regular assessment of respiratory function is crucial due to the frequent occurrence of respiratory complications and the progressive trend of Pompe disease [56,62,104]. The correct evaluation of respiratory muscle function is commonly achieved through measurements of FVC as well as maximal inspiratory (MIP) and expiratory (MEP) muscle pressures [105,106]. Before a noticeable decline in FVC occurs, weakness in the diaphragm can be identified through FVC measurements with the patient lying down or sitting [107]. Diaphragmatic weakness is detected in case of a reduction of ≥10% in FVC when measured in the supine position in comparison to the upright position; a decrease of ≥30% suggests severe weakness [106]. An observational study conducted on patients with late-onset Pompe disease reported decreased FVC in around 65% of cases. The remaining patients presented with respiratory muscle dysfunction, which was detected through a decline in FVC in the supine position or through MIP and MEP decrease [108,109].

Pompe disease patients with pulmonary involvement are mainly assisted through non-invasive mechanical ventilation. Current treatment relies on ERT. The effectiveness of ERT heavily relies on the patient’s age and their baseline residual pulmonary vital capacity [110]. The introduction of this treatment option significantly improved overall survival. However, evidence indicates that ventilatory support remains crucial, as most patients eventually experience respiratory insufficiency attributed to respiratory muscle failure [111].

Avalglucosidase alfa, a second-generation recombinant human GAA enzyme replacement therapy designed for increased cellular uptake and glycogen clearance, has been studied for long-term efficacy and safety in patients with late-onset Pompe disease (LOPD).

A multicenter open-label study using increasing doses assessed the safety, tolerability, pharmacokinetics, pharmacodynamics, and preliminary efficacy of repeat doses of avalglucosidase alfa (neoGAA) in individuals with late-onset Pompe disease (LOPD) [112]. Over a 24-week period, these patients received avalglucosidase alfa at doses of 5, 10, or 20 mg/kg every other week. Avalglucosidase alfa was generally well-tolerated, with no instances of death or life-threatening serious adverse events (SAEs). However, one Naïve patient withdrew from the study due to SAEs related to the study drug, experiencing respiratory distress and chest discomfort. Eight patients experienced infusion-associated reactions (IARs), most of which were non-serious and of mild-to-moderate intensity. Post-infusion, avalglucosidase alfa plasma concentrations decreased exponentially (with a half-life of approximately 1.0 h), with the area under the concentration-time curve (AUC) being 5–6 times higher in the 20 mg/kg group compared to the 5 mg/kg group. The pharmacokinetics were similar between the Switch and Naïve groups and remained consistent over time. Most patients had low baseline quadriceps muscle glycogen levels (approximately 6%), which generally did not change significantly throughout the study period. Exploratory efficacy measures, including pulmonary function and functional capacity, generally remained stable or showed improvement. The well-tolerated safety profile of avalglucosidase alfa and the promising preliminary efficacy results support further development of this treatment [112]. In conclusion, avalglucosidase alfa has been demonstrated to be well tolerated for up to 6.5 years in adult participants with LOPD, either naive to alglucosidase alfa or who had previously received alglucosidase alfa for ≥9 months [113].

## 4. Conclusions

Lung diseases (LSDs) are rare, multisystem disorders with a wide phenotypic heterogeneity. Pulmonary involvement may present with common respiratory symptoms such as dyspnea, cough, and recurrent infections. The challenge is to recognise milder forms, paying attention to historical and clinical data indicative of an underlying unique pathology. The pulmonologist can become part of the diagnostic pathway for these patients, often long before reaching a definitive diagnosis. Faster diagnosis and eventual specific treatment can change the natural history of LSDs.

## Figures and Tables

**Table 1:** MPS disorders classification with respective inheritance patterns, enzyme deficiency and involved genes [14].

MPS	Name	Inheritance Pattern	Enzyme Deficiency	Gene
I	Hurler, Hurler-Scheie, Scheie	Autosomal Recessive	α-L-iduronidase	*IDUA*
II	Hunter	X-linked Recessive	Iduronate-2-sulfatase	*IDS*
IIIA	Sanfilippo A	Autosomal Recessive	Heparan N-sulphatase	*SGHS*
IIIB	Sanfilippo B	Autosomal Recessive	*N*-acetyl-α-glucosaminidase	*NAGLU*
IIIC	Sanfilippo C	Autosomal Recessive	α-Glucosaminidase acetyltransferase	*HGSNAT*
IIID	Sanfilippo D	Autosomal Recessive	N-acetylglucosamine-6-sulfatase	*GNS*
IVA	Morquio A	Autosomal Recessive	Galactose-6-sulfate sulfatase	*GALNS*
IVB	Morquio B	Autosomal Recessive	β-galactosidase	*GLB1*
VI	Maroteauz-Lamy	Autosomal Recessive	*N*-acetylgalactosamine-4-sulfatase	*ARSB*
VII	Sly	Autosomal Recessive	β-D-glucuronidase	*GUS*
IX	Natowicz	Autosomal Recessive	Hyaluronoglucosaminidase 1	*HYAL1*
X	Arylsulfatase K deficiency	Autosomal Recessive	Arylsulfatase K	*ARSK*

**Table 2:** List of keywords adopted in the research strategy.

“Gaucher Disease”	**AND**	**“Lung disease” OR “Pulmonary involvement” OR “Respiratory symptoms” AND “Diagnosis” OR “Management” OR “Treatment” OR “Follow up”**
“Acid Sphingomyelinase deficiency”		
“Pompe disease”		
“Mucopolysaccharidosis”		

**Table 3:** Main non-respiratory clinical manifestations of the analysed LSDs.

Disease	System/Organ Involved	Clinical Manifestations
Gaucher disease	Liver and spleen	Hepato-splenomegaly
	Bone marrow	Anemia and thrombocytopenia with easy bruising and bleeding, bone pain
	Kidney	Glomerulopathy
	Musculoskeletal system	Osteopenia, growth restriction, fractures
	Central nervous system	Severe neurodegeneration typically in type 2, slow horizontal saccades typically in type 3
Acid sphingomyelinase deficiency	Liver and spleen	Hepato-splenomegaly, liver dysfunction
	Musculoskeletal system	Joint/limb pain and growth delay
	Eyes	Cherry-red maculae
	Heart	Left ventricular hypertrophy, conduction abnormalities and valvulopathies
	Central nervous system (infantile neurovisceral and chronic neurovisceral ASMD)	From mild hypotonia or hyporeflexia to loss of motor ability and mental impairment
Mucopolysaccharidoses	Musculoskeletal system	Skeletal deformities, joint rigidity, growth retardation, coarse faces, gibbus, laxity and flexibility of joints, even though hips and shoulder may be stiffer, hernias
	Central nervous system	Abnormal behavioral patterns, hypotonia, cognitive impairment, progressive cognitive decline, epilepsy
	Liver	Hepatomegaly
	Heart	Heart murmur, valvopathy
	Eye	Corneal clouding
	Ear	Hearing loss
Pompe disease	Musculoskeletal system	Hypotonia, myalgia, asthenia, exercise intolerance and elevated creatine kinase, osteoporosis, scoliosis, rigid spine syndrome
	Nervous System	Small-fiber neuropathy, sensorineural hearing loss
	Oral district	Macroglossia, lingual weakness with dysarthria and dysphagia, failure to thrive
	Heart	Cardiac hypertrophy or abnormal cardiac rhythm
	Pelvic district	Lower urinary tract (LUT) and anal sphincter involvement
	Gastrointestinal (GI) system	Impaired gastric function and GI motility

## Data Availability

Not applicable.
